# 409. A Phase 3, Randomized, Modified Double-blind Study Demonstrates Immunological Superiority of High-Dose (HD) Over Standard-Dose (SD) Inactivated Influenza Vaccine (IIV) in Adults 50 Through 64 Years of Age

**DOI:** 10.1093/ofid/ofaf695.016

**Published:** 2026-01-11

**Authors:** Rachna Gupta, Ashley Huang, Lisa Jackson, Stephanie Pepin, Tamala mallet Moore, Riyadh Obeng, Beth Kelly, Iris DeBruijn

**Affiliations:** Sanofi, Morristown, New Jersey; Sanofi, Morristown, New Jersey; Kaiser Permanente Washington Health Research Institute, Seattle, WA; Sanofi, Morristown, New Jersey; Sanofi, Morristown, New Jersey; Sanofi, Morristown, New Jersey; AstraZeneca, Gaithersburg, Maryland; Sanofi, Morristown, New Jersey

## Abstract

**Background:**

Adults 50-64 years have reduced immune response to SD influenza vaccines and higher morbidity associated with severe influenza than younger adults. IIV-HD (Fluzone^®^ HD) contains 4 times the antigen as IIV-SD, is preferentially recommended in the US for persons ≥65 years, and has a well-established safety profile. This study aimed to establish noninferiority (NI) and superiority of IIV- HD immune response compared to IIV-SD and assess safety in adults 50-64 years of age.

**Figure 1.**
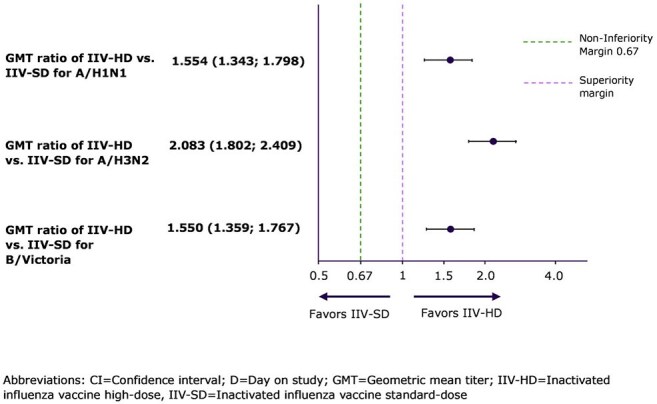
Forest plot - Post-vaccination GMT ratio and 95% CI of IIV-HD compared to IIV-SD at D29

**Table 1:**
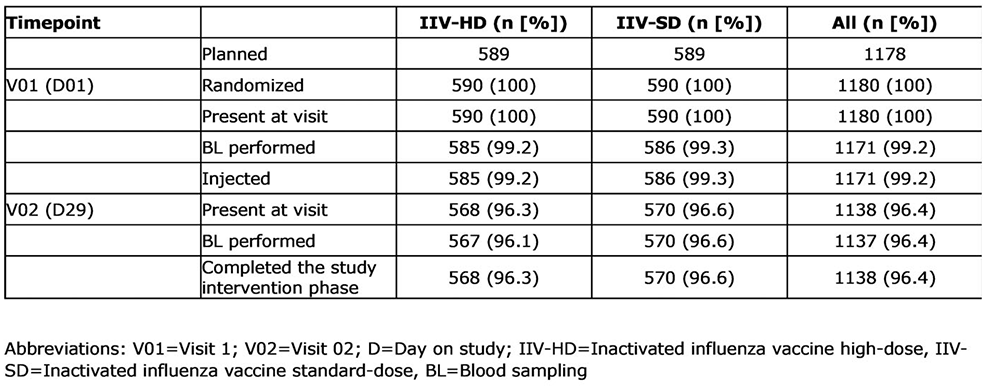
Disposition by randomized group

**Methods:**

A Phase 3, randomized, modified double-blind, active-controlled multicenter study (NCT06641180) was conducted in the US (Nov 2024 -Jun 2025). Participants 50-64 years old were randomized 1:1 to either IIV-HD or IIV-SD (Fluarix^®^, GSK) on Day (D) 01, followed for immunogenicity up to D29 and safety through 6 months. Primary objective was to evaluate NI of IIV-HD by ratio of geometric mean titer (GMT) and seroconversion rate (SCR), measured by hemagglutination inhibition assay (HAI), 28 days postvaccination. If NI was achieved for all 3 strains, IIV-HD superiority was assessed based on GMTs. If achieved, then superiority to a stringent threshold for at least one strain was tested.

**Figure:**
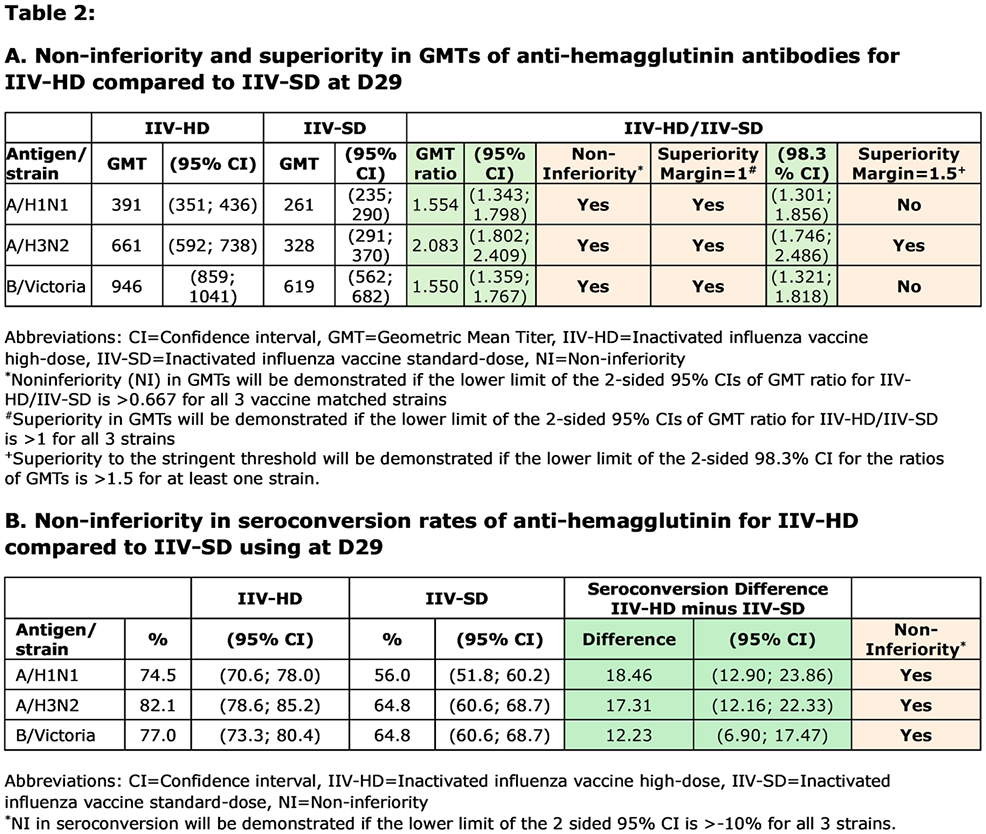

**Results:**

1171 participants were vaccinated; 1137 (96.4%) provided a blood sample at D01 and D29 (Table 1). Median age was 57 years; majority were female 54.9% and of white race 61.4%. Previous season influenza vaccination in 31.9% (IIV-HD) vs 33.4% (IIV-SD).

IIV-HD induced higher HAI GMTs and SCR than IIV-SD (Fig 1, Table 2). Coprimary immunogenicity endpoints of NI for HAI GMTs and SCR were achieved for all 3 vaccine matched strains.

IIV-HD also demonstrated a superior immune response vs IIV-SD by D29 GMT ratio (95% confidence interval [CI] lower limit >1) for all 3 strains. In addition, stringent threshold superiority was achieved for H3N2 for GMT ratio (98.3% CI lower limit >1.5).

The expected safety profile was confirmed in this age group through D29.

**Conclusion:**

In adults 50–64-years of age, IIV-HD demonstrated robust and superior immune responses to IIV-SD for all 3 influenza strains. IIV-HD met stringent superiority criteria for the H3N2 strain indicating HD H3N2 antibody levels are at least 50% higher than SD. These results support the safe and potential use of IIV-HD in this age group.

**Disclosures:**

All Authors: No reported disclosures

